# The Effects of Interaction between Climate Change and Land‐Use/Cover Change on Biodiversity‐Related Ecosystem Services

**DOI:** 10.1002/gch2.201800095

**Published:** 2019-05-06

**Authors:** Xinyue He, Jie Liang, Guangming Zeng, Yujie Yuan, Xiaodong Li

**Affiliations:** ^1^ College of Environmental Science and Engineering Hunan University Changsha 410082 P. R. China; ^2^ Key Laboratory of Environmental Biology and Pollution Control (Hunan University) Ministry of Education Changsha 410082 P. R. China; ^3^ Key Laboratory of Ecological Impacts of Hydraulic‐Projects and Restoration of Aquatic Ecosystem of Ministry of Water Resources Institute of Hydroecology Ministry of Water Resources and Chinese Academy of Sciences Wuhan 430079 P. R. China

**Keywords:** biodiversity, climate change, ecological responses, ecosystem services, land‐use/cover change

## Abstract

Climate change and land‐use/cover change (LUCC) are two major types of global environmental change. They are increasingly challenging the main objectives of ecosystem management, which are to provide ecosystem services sustainably to society and maintain biodiversity. However, a comprehensive understanding of how climate–land‐use change affects these primary goals of ecosystem management is still lacking. Here, a global literature review on the impacts of climate change and LUCC on ecosystem services related to biodiversity is presented. In this review, possible ecological responses at species, community, and ecosystem levels, and the effects of interaction mechanisms between climate change and LUCC on biodiversity‐related ecosystem services are identified. The results show possible effects on species facing climate change challenges through affecting distribution/range shifts, interspecific relations, richness, and abundance, and the impacts on biodiversity through increasing extinction rates, nutrient deposition, and habitat fragmentation under LUCC. Climate change may hinder the ability of species to deal with LUCC, and in turn LUCC could reduce resilience to climate change. Understanding of these interactions is necessary to address the increasing pressure on sustainable provisioning of ecosystem services under different climate and land‐use scenarios in the future.

## 1 Introduction

Biodiversity faces growing pressures from global changes, including climate change, habitat loss, invasive species, and pollution.^1^, ^2^ Although the abundance of species or size of communities is increasing or declining at regional and local scales, the Earth as a whole is experiencing substantial losses of biodiversity.^3^, ^4^ In any given area, climatic conditions are affecting broad landscapes as average temperature or precipitation may rise or fall, extreme values may become more intense, and the frequency of extreme climatic events may increase.^5^ Land‐use/cover change (LUCC) plays an important role in the climate system, ranging from the regional scale to the global scale.^6^ LUCC is affecting every biome on Earth and contributing to biodiversity loss. Correa et al.^7^ found that direct and indirect LUCC are major pressures negatively impacting biodiversity through biofuel production, particularly when ecosystems with high biodiversity values (e.g., tropical and subtropical forests and native grasslands) are transformed into biofuel plantations. As the impacts of climate change and LUCC intensify and interact in the coming decades, the threats to biodiversity may be amplified, thereby increasing rates of population decline and extinction risk.^8^

These changes are important natural and anthropogenic drivers of ecosystem dynamics, and strongly modulate the structure and functioning of ecosystems.^9^ Changing environment might thus considerably alter the ecosystems, with potentially far‐reaching impacts on their biodiversity and capacity to provide ecosystem services to society. It is necessary to utilize their beneficial effects on biodiversity aiming to emulate changes in ecosystem management, instead of strict avoidance of changes due to negative effects on selected ecosystem services.^10^ When multiple targets need to be realized within a specific landscape, changes can be expected to have both positive and negative impacts on possible objectives of ecosystem management. In recent decades, not only global changes have increased recently, but also the range and demand for ecosystem services have been growing steadily. Therefore, how to balance these positive and negative impacts on in the particular context of changing environment will be a key challenge for future ecosystem management.

**Figure 1:**
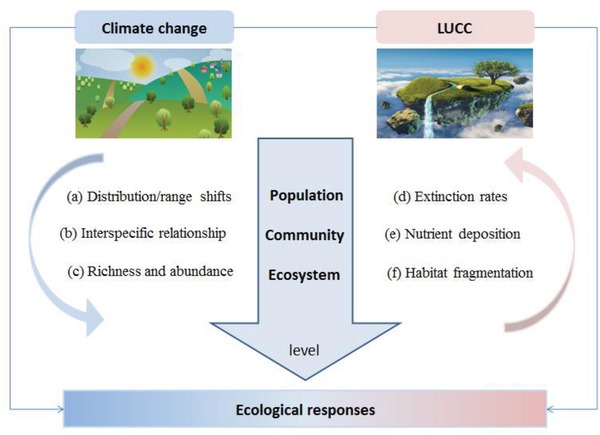
The framework of ecological responses triggered by climate change and LUCC on different levels in this review.

With the aim to provide ecosystem services to society while fostering biodiversity, ecosystem management requires a comprehensive understanding of the impacts of climate–land‐use change. Yet only a handful of studies address multiple interacting threats when prioritizing management actions for conservation, so the benefits of abating a single threat may be overestimated.^11^ In this paper, we focused on two major global change drivers, namely climate change and LUCC (**Figure** **1**), and attempted to describe their interactive effects on biodiversity‐related ecosystem services. Our overall objectives were to 1) elaborate ecological responses to future climate–land‐use change for selected indicators of ecosystem services and biodiversity; 2) discuss how climate change and LUCC interact to affect biodiversity‐related ecosystem services via a quantitative meta‐analysis; and 3) identify the current needs and the future priorities in ecosystem management.

## 2 Material and Methods

### 2.1 Data Collection

**Figure 2:**
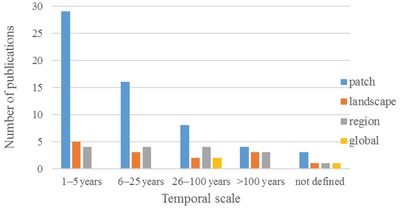
Temporal and spatial scales of observations regarding climate–land‐use change impact on ecosystem services and biodiversity reported in peer‐reviewed publications included in the analysis. “Not defined” means undefined temporal scale in the study.

In order to find the best available knowledge reported by the scientific community, we focused on peer‐reviewed academic literature from 2000 to October 2018. These publications were identified from the Institute for Scientific Information (ISI) Web of Science using the following search terms: biodiversity or species richness or habitat quality or indices of diversity, ecosystem services or ecosystem function, land use, and climate change. Specially, reviews and syntheses were excluded in order to avoid double counting and the potential transfer of artifacts or errors from one review to the next. For each of these studies, we collected information on geographical location and spatial and temporal scales. To be specific, ecological effects were grouped into four different time horizons: short term (1–5 years), midterm (6–25 years), long term (26–100 years), and very long term (>100 years); at the spatial scale, they were divided into four parts: patch (1–100 ha), landscape (101–100 000 ha), region (>100 000 ha), and global (**Figure** **2**).

### 2.2 Data Analysis

We analyzed our literature‐derived database of climate–land‐use impact on biodiversity‐related ecosystem services in two steps. In the first step, we assessed ecological responses to climate change and LUCC with indicators of ecosystem services and biodiversity. In the second step, in order to understand the interactive effect, we conducted a meta‐analysis based on quantitative information on interaction mechanisms between climate change and LUCC.

## 3 Results

### 3.1 Ecological Responses

In recent years, the responses of species to climate change have received increasing attention at local, regional, and global scales.^12^ Biodiversity is often influenced by climatic variations including changes in temperature, precipitation, evaporation, radiant flux, carbon emissions, and the increasingly frequent extreme climatic events. As climate continues to change at an increasing rate, species and ecosystems can be expected to respond accordingly, though their response depends on multiple factors from species, community, and ecosystem levels.^13^ These will affect distribution and range shifts, interspecific relations, and abundance.

Most obviously, climate change has the potential to affect the distribution and range of all species,^14^ so many studies focus on current geographic distributions to inform predicted shifts under various climate change scenarios.^15^ However, a major source of uncertainty is the spatial mismatch between the range of species and the scale at which climate data are collected and modeled.^16^ Species, and their genetic diversity, are able to adapt to climate change when the changing ecological conditions are suitable.^17^ Climate‐induced range shifts of species have been confirmed along altitudinal and latitudinal gradients where dispersal mechanisms and resource availability allowed such shifts.^18^, ^19^ Even so, the shift has not been fast enough to keep pace with climate change for numerous species, especially plants whose capacity to move tend to be more conservative than the more mobile animals. Since species will vary in their capacity to move, the new communities being established in response to climate change are likely to be novel and dynamic until the climate returns to a sort of stability at some point in the future.^20^, ^21^ Subtle impacts on basic biological parameters of individual species may bring about great changes in community's composition and structure.^22^ Variation in interaction outcomes often occurs along gradients like temperature, seasonality, and phenology for plants.^23^

Biodiversity also plays an important role in the provision of ecosystem functions and services.^24^, ^25^, ^26^ The positive correlation between biodiversity and ecosystem services is supported by a substantial literature.^27^ Specially, from 2010 onward, the field of ecosystem services related to biodiversity has grown rapidly. Approximately 80% of studies were conducted in either North America or Europe, and the remaining 20% of studies occurred in Asia, Oceania, and Africa. The functional consequences of species loss may be mitigated or exacerbated by the simultaneous effect of species' richness and relative abundance.^28^ For example, cold regions that become warmer and arid regions that receive increased precipitation are both likely to support a greater diversity of species. In most ecosystem processes, the influence of biodiversity is nonlinear, and change may accelerate as biodiversity loss increases.^29^, ^30^

Compared with climate change, the future effects of LUCC on biodiversity are receiving less attention. Yet LUCC has a huge impact on agricultural production, greenhouse gases, recreation, urban green spaces, and species diversity.^31^ With climate change, species show adaptive responses to complex land‐use scenarios. LUCC has been used as a basis for forecasting future changes in suitable habitats, detecting future invasive species, estimating potential future extinctions in biodiversity hotspots, and highlighting the restricted potential for future expansion of protected areas worldwide.^32^

At the species level, the most common response to LUCC is increasing extinction rates, so conservation actions need to be prioritized based on species' extinction risk. Species with long generation times and populations near their extinction threshold are most likely to have an extinction debt,^33^ and small populations have a higher extinction probability than large populations.^34^ Hull et al.^35^ assessed the magnitude of the current biodiversity crisis relative to past crises by extrapolating extinction rates. Usually, dominant species in a habitat are considered to be at low extinction risk, and species with poor ability to adapt are more likely to become threatened or even extinct when the area available is reduced by habitat destruction. As a consequence, efforts to slow the rate of habitat loss have focused foremost on rarer species, which, by definition, face the greatest extinction threats.^36^ Species at higher trophic levels are more susceptible to extinction resulting from habitat fragmentation and severe disturbances. If future locations of land use do not coincide with other essential resources like soil type and food resources, extinction risks might be higher than projected.^37^ Responses by individual species to environmental changes are not isolated, but take place in the context of communities and ecosystems that provide nutrients for growth and reproduction. Increased nutrient deposition as a consequence of land‐use change alters soil fertility and may have positive indirect effects on soil biota by improving the quality of resources that plants return to the soil. The plant nutrient cycling and nutrient supply processes generally drive short‐term temporal dynamics of soil biota.^38^, ^39^

The process of land‐use conversion is usually accompanied by habitat fragmentation, which may affect ecosystem function by decreasing patch size or lengthen distance between patches.^40^ At the ecosystem scale, increasing the size or number of patches may improve resilience by increasing the effective size of populations and decreasing edge effects. In ecosystems with extensive fragmented areas, patch size and isolation effects will intensify the effect of habitat loss. Consequently, the loss of species or decline in population size will be greater than expected from habitat loss alone. In such a situation, larger patches and more connectivity may be required to maintain populations.^41^ The existence of a species in a patch depends on the patch size and connectivity between different patches, as well as the adjacent habitat. When neighboring habitats help create new patches, the diversity of total species in a given ecosystem may increase because new species may be found in these new habitats. Habitat generalists can survive in very small patches because they can also utilize resources in the surroundings.^42^ In summary, the effects of habitat fragmentation on biodiversity are diverse and significant, with different ways of measuring these effects and thereby drawing various conclusions regarding both the magnitude and direction of its effects.^43^, ^44^, ^45^, ^46^

### 3.2 Interaction Mechanisms

**Figure 3:**
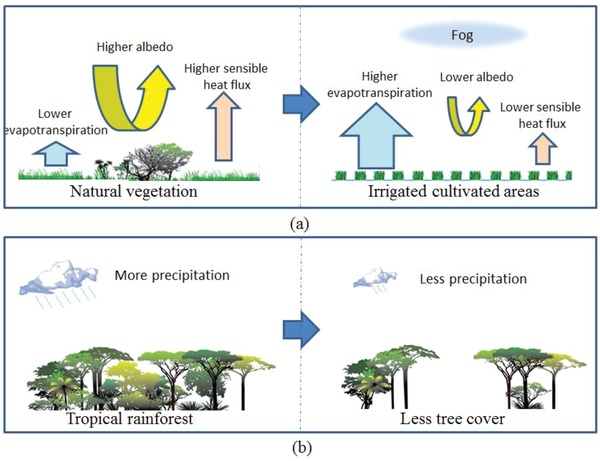
The representative climatic impacts of LUCC based on the literature reviewed. a) Changes in surface climate after introduction of irrigated agriculture. b) Influence of historic deforestation on patterns of precipitation.

Climate change and LUCC interact to affect biodiversity through a wide range of pathways. Climate change can hinder the ability of species to deal with LUCC, and LUCC can reduce resilience to climate change.^47^ A meta‐analysis on interactions between habitat loss effects and climate concluded that the effects of habitat loss were greatest in areas with higher mean temperatures and decreased mean precipitation,^48^ and therefore LUCC must be a major concern for those working to address climate change (**Figure** **3**). Some interactive mechanisms that how climate change interacts with LUCC in several representative pathways and ultimately affects biodiversity are discussed in the following sections.

#### 3.2.1 Impacts of Extreme Weather Events on Habitat Adaptation

Extreme climatic events, such as droughts, floods, and hurricanes, are projected to become more frequent and intense in the coming decades.^49^, ^50^ The potential adaptation to habitat configuration is likely to be influenced by both the frequency and severity of these extreme climatic events.^51^ For example, a changing climate may push a plant species closer to the edge of its fundamental niche space, reducing its competitive ability and allowing other plant species to become dominant, a process which is exacerbated under extreme weather conditions. Increasing incidence of extreme events may also lead to cold‐associated species being replaced by warm‐adapted species in some places. But most research on the effects of climate change has focused primarily on the consequences of increased average temperatures rather than on the impacts of extreme climatic events.

Indirect sharp modifications of habitats will give populations less time to recover from extreme climatic events, for example, by reinforcing their negative impacts by decreasing the ability of species to recover from environmental perturbation. Substantial examples emphasize how important it is to consider the impacts of extreme climatic events in the context of LUCC. The responses to increasing extreme events influence plant functioning across spatial and temporal scales, and plant–water relations seem to be very vulnerable to extremes; flooding may have stronger impacts on physiological processes than changing mean climate.^52^ Meanwhile, past extreme conditions have shaped the current ecosystems and may also foster adaptation.

#### 3.2.2 Influences of Climate Mitigation Policies on Land‐Use Decisions

Climate change also affects land use through changes to socioeconomic regimes and adaptation policy. To a great extent, how society addresses climate change will affect biodiversity when mitigation policies reduce direct climate change impacts. Meanwhile, these policies will influence land‐use decisions which could have either positive or negative influences on habitat for a large number of species. The mismatch between climate change mitigation and conserving biodiversity is most apparent in the tropics. Sometimes, efforts to protect carbon‐storing trees to mitigate climate change may result in conserving only an empty forest.^53^ It is therefore critical to understand the consequences of interactions for biodiversity and considering the prioritization of multiple conservation actions.^54^

In a region mostly with intact ecosystems, it is wise to prevent anthropogenic habitat degradation, and thereby maintain its intrinsic stability. In the process of habitat restoration, allowing species to migrate with global warming can help increase the resilience of fragmented landscapes in the face of climate change. As climate change mitigation effort increases, loss of natural vegetative cover that directly resulted from LUCC generally declines. The implementation of measures to mitigate climate change could simultaneously prevent habitat loss driven by LUCC in biodiversity hotspots. For ecological communities, whose mobility is limited, such as freshwater communities, buffer strips can be established to minimize damage from agricultural runoff.^55^ Other policy responses may include installation of climate refuges,^56^ translocation of imperiled species,^57^ and construction or restoration of riparian habitats.^58^ Therefore, future action plans to address the twin objectives of climate mitigation and biodiversity conservation should consider habitat protection as a joint mitigation–conservation priority.

#### 3.2.3 Effects of Rising CO_2_ Concentrations on the Macroclimate

LUCC accounts for 12.5% of anthropogenic carbon emissions.^59^ Related land‐use variables such as changing vegetation types, enhanced rates of decomposition, and substantial losses of forest are closely linked to rising CO_2_ concentrations. Complex changes related to carbon emissions have included extensive changes in the quantity, structure, and spatial pattern of land‐use types, particularly in the soil food web. The intensity of land use exerts a significant direct impact on soils, and they are also subject to indirect impacts arising from human activities such as acid deposition and heavy metal pollution.^60^, ^61^ On the other hand, many plants benefit from increase in CO_2_ through promoting photosynthesis and decreasing water stress.^62^

**Figure 4:**
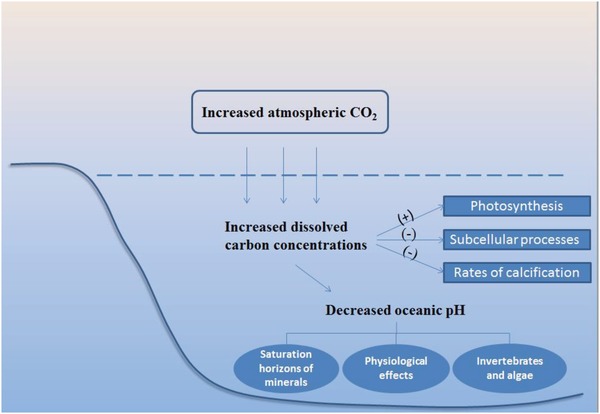
Important physical and chemical changes associated with CO_2_ enrichment in the marine system.

In coastal marine systems, early life stages of marine organisms are potentially vulnerable to the stressors associated with global climate change. Identifying general patterns across species and response variables is challenging,^63^ but it is clear that rates of calcification in corals and coralline red algae are likely to drop under increased CO_2_.^64^ As dissolved carbon concentrations increase, a cascade of physical and chemical changes will appear (**Figure** **4**). Short‐term experimental results indicate that rising CO_2_ concentrations can lead to decreases in subcellular processes such as protein synthesis and ion exchange. In the long run, the anticipated acidification of the oceans could be expected to have severe negative impacts on many marine invertebrates and algae. Considering that the expected pH drop may be unprecedented, deeper studies on the biotic effects of pH change are urgently needed.

#### 3.2.4 Changes of Microclimatic Gradients Induced by Habitat Modification

Global biodiversity is changing with climate gradient, and land‐use change can modify microclimatic gradients. At any given time, the spatial arrangement of habitats may influence landscape processes such as functional connectivity, edge effects, and ecological integrity.^65^ These processes are particularly important in landscapes that are intensively managed, and natural or seminatural habitats are highly fragmented. Greater variation in spatial heterogeneity tends to a wider environmental tolerance, which may improve resilience to climate change. In general, increasing land‐use heterogeneity has limited potential to offset negative effects of intensification. In other words, even moderate increases in local land‐use intensity can cause biotic homogenization. Mountainous regions tend to be heterogeneous, including great topographic and altitudinal diversity, so that species may be better able to escape the effects of climate change vertically.^66^ However, in a landscape with low heterogeneity, species are forced to seek adaptive climatic conditions for longer distance movement. For instance, tropical ecosystems often have a shallow latitudinal gradient in temperature that makes it harder for organisms to shift ranges poleward compared with temperate organisms.^67^ By adopting the target of maintaining spatial heterogeneity at the landscape scale, biodiversity conservation strategies will become more effective.

Disadvantageous landscape structure may severely hinder species movement and their ability to track climate envelopes. Species may shift downslope or upslope as a direct consequence of habitat modification, either following permanent habitat changes such as recreational activities and management practices, or owing to other local changes in habitat suitability. Species with greater dispersal ability, higher reproductive rate, and ecological generalization are better able to expand into new regions and establish new populations, so they tend to have larger geographical ranges. Conversely, species with low adaptability and dispersal capacity will have difficulty finding appropriate habitats to occupy, ultimately leading to increased extinction rates. In reality, the process of migration itself is likely to be dangerous because migrating individuals may be forced to move to unsuitable habitats or be exposed to predators during flight.^68^

## 4 Discussion and Conclusions

Previous studies have provided valuable information on the role of biodiversity in the provision of ecosystem service from a theoretical perspective, ranging from the relationship between functional traits and ecosystem services to the pathway that how biodiversity influences ecosystem functions and their ability to provide ecosystem services.^69^, ^70^ In this study, we went a step further to build up the scientific knowledge based on ecosystem services, their biodiversity attributes, the direction and strength of evidence for these relationships, and the influence of abiotic factors. Our results would examine whether habitat management involving manipulation of vegetation can be used to offset the adverse impacts of changes in temperature, precipitation, and extreme climatic events on biodiversity. To minimize global biodiversity loss, researchers and habitat managers need to improve their understanding of the biological effects of climate and LUCC, and how these effects can be mitigated through management interventions. Ecological responses to climate change and LUCC are challenging and not always easy to detect, not least because the responses of organisms may vary among species or functional groups,^71^ geographic locations, spatial extent,^72^ and as a function of time since fragmentation of their habitat.^73^ Environmental conditions are fluctuating continuously and population responses to global change may be altered by changing environmental variance in the mean environment.^74^

The combination of impacts on biodiversity is likely to be nonlinear rather than a simple superposition under different climate and land‐use scenarios. Effects may be additive, synergistic, or antagonistic when two variables work together. On one hand, future climate scenarios are filled with uncertainty globally. Carroll et al.^75^ demonstrated that change in climatic velocities, which were derived from different general circulation models and emissions pathways, was less than the variation among other environmental diversity indicators. As information on the direction and speed of climate change becomes more reliable, it may be possible for planners to assess priorities on the basis of replaceable indicators that can be applied to a highly variable region. On the other hand, land use is driven by both natural (climate change, soil condition, vegetation succession, etc.) and socioeconomic factors (e.g., demographic change, poverty status, technological progress, and political and economic structure) with complex feedbacks. The relationship of driving forces is often quantified by the combination of conceptual models and mathematical model.^76^ However, the main driving force remains uncertain due to the variability of regional characteristics.^77^ If the negative impacts of land‐use change cannot be addressed, then the options for dealing with climate change are greatly reduced. At a broad global scale, the socioeconomic system to meet increased human population size, growing food demands, and changes in urban planning policies is set to continue. In this regard, greater attention needs to be given to the interacting effects of other anthropogenic stressors such as movements of people, hunting, poaching, and illegal wildlife trading.

Crucially, the threats to biodiversity are stimulating scientists to make definitive predictions about the impacts of this process. Current estimates are diverse, depending on the modeling approaches, taxonomic groups, biodiversity loss metrics (e.g., species richness, vulnerability, or extinction rate), and spatial scales and time periods considered. Some models have considered just a single taxon or endangered rare species, but these results are not representative. Many focus on specific hotspots or on the local area of the researcher. It is crucial to begin developing spatially explicit global forecasts that go beyond the demographic dimension of the species concerned. Biodiversity is lost from fragmented habitats, but also exerts a far‐reaching influence on the larger landscape. It would be helpful to improve biodiversity monitoring by using available remote sensing (RS) applications that could be expanded into national indicators.^78^ Rapidly emerging new technologies from drones to airborne laser scanning and new satellite sensors providing imagery with very high resolution open a whole new world of opportunities for monitoring biodiversity at low cost. Furthermore, solutions to biodiversity loss may come from some standardization of future research (e.g., of taxonomic groups, methods, time horizon, and scale). This might help decrease uncertainty at the expense of the breadth of knowledge and bring much required innovation to this field. A major near‐term target is to substantially improve understanding, predictive capacity, and reactive potential that will help researchers, policy makers, and conservation practitioners to better minimize risks and exploit opportunities provided by climate–land‐use change interactions.

## Conflict of Interest

The authors declare no conflict of interest.

## References

[1] D. P. Tittensor , M. Walpole , S. L. Hill , D. G. Boyce , G. L. Britten , N. D. Burgess , S. H. Butchart , P. W. Leadley , E. C. Regan , R. Alkemade , Science 2014, 346, 241. 25278504 10.1126/science.1257484

[2] B. Gallardo , M. Clavero , M. I. Sánchez , M. Vilà , Global Change Biol. 2016, 22, 151. 10.1111/gcb.13004 26212892

[3] D. A. Wardle , Science 2011, 332, 1273. 21659595 10.1126/science.1197479

[4] P. Yoan , B. Laurent , H. Joakim , Ó. Péter , A. Catherine , B. Markus , B. Rienkjan , D. B. Luc , F. Marc , G. Ulf , Conserv. Biol. 2010, 24, 101.20121845

[5] R. A. Garcia , M. Cabeza , C. Rahbek , M. B. Araújo , Science 2014, 344, 1247579. 24786084 10.1126/science.1247579

[6] R. Mahmood , R. A. Pielke Sr. , K. G. Hubbard , D. Niyogi , P. A. Dirmeyer , C. Mcalpine , A. M. Carleton , R. Hale , S. Gameda , A. Beltrán‐Przekurat , Int. J. Climatol. 2014, 34, 929.

[7] D. F. Correa , H. L. Beyer , H. P. Possingham , S. R. Thomas‐Hall , P. M. Schenk , Renewable Sustainable Energy Rev. 2017, 74, 1131.

[8] J. L. Payne , A. M. Bush , N. A. Heim , M. L. Knope , D. J. Mccauley , Science 2016, 353, 1284. 27629258 10.1126/science.aaf2416

[9] M. G. Turner , Ecology 2010, 91, 2833. 21058545 10.1890/10-0097.1

[10] M. Overbeck , M. Schmidt , For. Ecol. Manage. 2012, 266, 115.

[11] N. A. Auerbach , K. A. Wilson , A. I. T. Tulloch , J. R. Rhodes , J. O. Hanson , H. P. Possingham , Conserv. Biol. 2015, 29, 1626. 26171646 10.1111/cobi.12551

[12] C. Bellard , C. Bertelsmeier , P. Leadley , W. Thuiller , F. Courchamp , Ecol. Lett. 2012, 15, 365. 22257223 10.1111/j.1461-0248.2011.01736.x PMC3880584

[13] K. R. Jones , J. E. M. Watson , H. P. Possingham , C. J. Klein , Biol. Conser. 2016, 194, 121.

[14] J. T. Kerr , A. Pindar , P. Galpern , L. Packer , S. G. Potts , S. M. Roberts , P. Rasmont , O. Schweiger , S. R. Colla , L. L. Richardson , Science 2015, 349, 177. 26160945 10.1126/science.aaa7031

[15] P. D. Henne , M. Bigalke , U. Büntgen , D. Colombaroli , M. Conedera , U. Feller , D. Frank , J. Fuhrer , M. Grosjean , O. Heiri , J. Luterbacher , A. Mestrot , A. Rigling , O. Rössler , C. Rohr , T. Rutishauser , M. Schwikowski , A. Stampfli , S. Szidat , J.‐P. Theurillat , R. Weingartner , W. Wilcke , W. Tinner , Reg. Environ. Change 2018, 18, 205.

[16] K. A. Potter , W. H. Arthur , S. Pincebourde , Global Change Biol. 2013, 19, 2932. 10.1111/gcb.12257 23681970

[17] T. P. Dawson , S. T. Jackson , J. I. House , I. C. Prentice , G. M. Mace , Science 2011, 332, 53. 21454781 10.1126/science.1200303

[18] A. L. Angert , L. G. Crozier , L. J. Rissler , S. E. Gilman , J. J. Tewksbury , A. J. Chunco , Ecol. Lett. 2011, 14, 677. 21535340 10.1111/j.1461-0248.2011.01620.x

[19] I. Chen , J. K. Hill , R. Ohlemüller , D. B. Roy , C. D. Thomas , Science 2011, 333, 1024. 21852500 10.1126/science.1206432

[20] P. D. Frenne , F. Rodríguezsánchez , D. A. Coomes , L. Baeten , G. Verstraeten , M. Vellend , M. Bernhardtrömermann , C. D. Brown , J. Brunet , J. Cornelis , Proc. Natl. Acad. Sci. USA 2013, 110, 18561.24167287

[21] R. Bertrand , J. Lenoir , C. Piedallu , G. Riofrío‐Dillon , R. P. De , C. Vidal , J. C. Pierrat , J. C. Gégout , Nature 2011, 479, 517. 22012261 10.1038/nature10548

[22] G. R. Walther , Philos. Trans. R. Soc. London 2010, 365, 2019. 20513710 10.1098/rstb.2010.0021 PMC2880129

[23] S. A. Chamberlain , J. L. Bronstein , J. A. Rudgers , Ecol. Lett. 2014, 17, 881. 24735225 10.1111/ele.12279

[24] E. M. Bennett , W. Cramer , A. Begossi , G. Cundill , S. Díaz , B. N. Egoh , I. R. Geijzendorffer , C. B. Krug , S. Lavorel , E. Lazos , Curr. Opin. Environ. Sustainability 2015, 14, 76.

[25] G. M. Mace , K. Norris , A. H. Fitter , Trends Ecol. Evol. 2012, 27, 19. 21943703 10.1016/j.tree.2011.08.006

[26] S. Soliveres , F. van der Plas , P. Manning , D. Prati , M. M. Gossner , S. C. Renner , F. Alt , H. Arndt , V. Baumgartner , J. Binkenstein , Nature 2016, 536, 456. 27533038 10.1038/nature19092

[27] T. Newbold , L. N. Hudson , S. L. Hill , S. Contu , I. Lysenko , R. A. Senior , L. Börger , D. J. Bennett , A. Choimes , B. Collen , Nature 2015, 520, 45. 25832402 10.1038/nature14324

[28] L. A. Garibaldi , I. Steffandewenter , R. Winfree , M. A. Aizen , R. Bommarco , S. A. Cunningham , C. Kremen , L. G. Carvalheiro , L. D. Harder , O. Afik , Science 2013, 339, 1608. 23449997 10.1126/science.1230200

[29] B. J. Cardinale , D. S. Srivastava , J. E. Duffy , J. P. Wright , A. L. Downing , M. Sankaran , C. Jouseau , Nature 2006, 443, 989. 17066035 10.1038/nature05202

[30] A. S. Mori , K. P. Lertzman , L. Gustafsson , J. Appl. Ecol. 2016, 54, 12.

[31] I. J. Bateman , M. Termansen , Science 2013, 341, 45. 23828934 10.1126/science.1234379

[32] E. V. Moran , J. M. Alexander , Ecol. Lett. 2014, 17, 637. 24612028 10.1111/ele.12262

[33] M. Kuussaari , R. Bommarco , R. K. Heikkinen , A. Helm , J. Krauss , R. Lindborg , E. Ockinger , M. Pärtel , J. Pino , F. Rodà , Trends Ecol. Evol. 2009, 24, 564. 19665254 10.1016/j.tree.2009.04.011

[34] D. T. Flockhart , J. B. Pichancourt , D. R. Norris , T. G. Martin , J. Anim. Ecol. 2015, 84, 155. 24903085 10.1111/1365-2656.12253

[35] P. M. Hull , S. A. Darroch , D. H. Erwin , Nature 2015, 528, 345. 26672552 10.1038/nature16160

[36] K. J. Gaston , Science 2010, 327, 154. 20056880 10.1126/science.1182818

[37] C. D. Thomas , A. Cameron , R. E. Green , M. Bakkenes , L. J. Beaumont , Y. C. Collingham , B. F. Erasmus , M. F. De Siqueira , A. Grainger , L. Hannah , Nature 2004, 427, 145. 14712274 10.1038/nature02121

[38] R. D. Bardgett , W. H. van der Putten , Nature 2014, 515, 505. 25428498 10.1038/nature13855

[39] D. L. Huang , G. M. Zeng , C. L. Feng , S. Hu , X. Y. Jiang , L. Tang , F. F. Su , Y. Zhang , W. Zeng , H. L. Liu , Environ. Sci. Technol. 2008, 42, 4946. 18678031 10.1021/es800072c

[40] L. Fahrig , J. Biogeogr. 2013, 40, 1649.

[41] E. Öckinger , O. Schweiger , T. O. Crist , D. M. Debinski , J. Krauss , M. Kuussaari , J. D. Petersen , J. Pöyry , J. Settele , K. S. Summerville , Ecol. Lett. 2010, 13, 969. 20482577 10.1111/j.1461-0248.2010.01487.x

[42] D. A. Driscoll , S. C. Banks , P. S. Barton , D. B. Lindenmayer , A. L. Smith , Trends Ecol. Evol. 2013, 28, 605. 23883740 10.1016/j.tree.2013.06.010

[43] J. L. McCune , J. Appl. Ecol. 2016, 53, 1871.

[44] E. M. Simons‐Legaard , D. J. Harrison , K. R. Legaard , J. Appl. Ecol. 2016, 53, 1260.

[45] S. Saura , O. Bodin , M.‐J. Fortin , J. Appl. Ecol. 2014, 51, 171.

[46] M. G. E. Mitchell , A. F. Suarez‐Castro , M. Martinez‐Harms , M. Maron , C. McAlpine , K. J. Gaston , K. Johansen , J. R. Rhodes , Trends Ecol. Evol. 2015, 30, 190. 25716547 10.1016/j.tree.2015.01.011

[47] W. N. Adger , K. Brown , D. R. Nelson , F. Berkes , H. Eakin , C. Folke , K. Galvin , L. Gunderson , M. Goulden , K. O'Brien , Wiley Interdiscip. Rev.: Clim. Change 2011, 2, 757.

[48] C. S. Mantyka‐Pringle , T. G. Martin , J. R. Rhodes , Global Change Biol. 2013, 19, 1642.

[49] P. A. Stott , N. Christidis , F. E. L. Otto , Y. Sun , J.‐P. Vanderlinden , G. J. van Oldenborgh , R. Vautard , H. von Storch , P. Walton , P. Yiou , F. W. Zwiers , Wiley Interdiscip. Rev.: Clim. Change 2016, 7, 23. 26877771 10.1002/wcc.380 PMC4739554

[50] M. Leonard , S. Westra , A. Phatak , M. Lambert , B. V. D. Hurk , K. Mcinnes , J. Risbey , S. Schuster , D. Jakob , M. Stafford‐Smith , Wiley Interdiscip. Rev.: Clim. Change 2014, 5, 113.

[51] G. W. Gilchrist , Am. Nat. 1995, 146, 252.

[52] C. P. O. Reyer , S. Leuzinger , A. Rammig , A. Wolf , R. P. Bartholomeus , A. Bonfante , F. d. Lorenzi , M. Dury , P. Gloning , R. A. Jaoudé , Global Change Biol. 2013, 19, 75. 10.1111/gcb.12023 PMC3857548 23504722

[53] T. Caro , M. B. Mulder , Nature 2016, 537, 617. 10.1038/537617d 27680932

[54] T. H. Oliver , M. D. Morecroft , Wiley Interdiscip. Rev.: Clim. Change 2014, 5, 317.

[55] L. Castello , M. N. Macedo , Global Change Biol. 2016, 22, 990. 10.1111/gcb.13173 26700407

[56] L. P. Shoo , D. H. Olson , S. K. Mcmenamin , K. A. Murray , M. Van Sluys , M. A. Donnelly , D. Stratford , J. Terhivuo , A. Merino‐Viteri , S. M. Herbert , J. Appl. Ecol. 2011, 48, 487.

[57] M. W. Schwartz , T. G. Martin , Ann. N. Y. Acad. Sci. 2013, 1286, 15. 23574620 10.1111/nyas.12050

[58] R. M. Kreiling , J. P. Schubauer‐Berigan , W. B. Richardson , L. A. Bartsch , P. E. Hughes , J. C. Cavanaugh , E. A. Strauss , J. Environ. Qual. 2013, 42, 573. 23673850 10.2134/jeq2012.0248

[59] R. A. Houghton , J. I. House , J. Pongratz , G. R. van der Werf , R. S. DeFries , M. C. Hansen , C. Le Quere , N. Ramankutty , Biogeosciences 2012, 9, 5125.

[60] P. Smith , J. I. House , M. Bustamante , J. Sobocká , R. Harper , G. Pan , P. C. West , J. M. Clark , T. Adhya , C. Rumpel , Global Change Biol. 2016, 22, 1008. 10.1111/gcb.13068 26301476

[61] L. Tang , G. M. Zeng , G. L. Shen , Y. P. Li , Y. Zhang , D. L. Huang , Environ. Sci. Technol. 2008, 42, 1207. 18351094 10.1021/es7024593

[62] C. D. G. Harley , A. R. Hughes , K. M. Hultgren , B. G. Miner , C. J. B. Sorte , C. S. Thornber , L. F. Rodriguez , L. Tomanek , S. L. Williams , Ecol. Lett. 2006, 9, 228. 16958887 10.1111/j.1461-0248.2005.00871.x

[63] R. Przeslawski , M. Byrne , C. Mellin , Global Change Biol. 2015, 21, 2122. 10.1111/gcb.12833 25488061

[64] K. L. Nash , N. A. J. Graham , S. Jennings , S. K. Wilson , D. R. Bellwood , J. Appl. Ecol. 2016, 53, 646.

[65] M.‐A. Villard , J. P. Metzger , J. Appl. Ecol. 2014, 51, 309.

[66] S. R. Loarie , P. B. Duffy , H. Hamilton , G. P. Asner , C. B. Field , D. D. Ackerly , Nature 2009, 462, 1052. 20033047 10.1038/nature08649

[67] R. K. Colwell , G. Brehm , C. L. Cardelús , A. C. Gilman , J. T. Longino , Science 2008, 322, 258. 18845754 10.1126/science.1162547

[68] J. W. Chapman , D. R. Reynolds , K. Wilson , Ecol. Lett. 2015, 18, 287. 25611117 10.1111/ele.12407

[69] B. J. Cardinale , J. E. Duffy , A. Gonzalez , D. U. Hooper , C. Perrings , P. Venail , A. Narwani , G. M. Mace , D. Tilman , D. A. Wardle , Nature 2012, 486, 59. 22678280 10.1038/nature11148

[70] F. D. Bello , S. Lavorel , S. Díaz , R. Harrington , J. H. C. Cornelissen , R. D. Bardgett , M. P. Berg , P. Cipriotti , C. K. Feld , D. Hering , Biodiversity Conserv. 2010, 19, 2873.

[71] D. Vetter , M. M. Hansbauer , Z. Végvári , I. Storch , Ecography 2011, 34, 1.

[72] A. C. Smith , L. Fahrig , C. M. Francis , Ecography 2011, 34, 103.

[73] T. Callens , P. Galbusera , E. Matthysen , E. Y. Durand , M. Githiru , J. R. Huyghe , L. Lens , Mol. Ecol. 2011, 20, 1829. 21492264 10.1111/j.1365-294X.2011.05028.x

[74] C. R. Lawson , Y. Vindenes , L. Bailey , M. van de Pol , Ecol. Lett. 2015, 18, 724. 25900148 10.1111/ele.12437

[75] C. Carroll , D. R. Roberts , J. L. Michalak , J. J. Lawler , S. E. Nielsen , D. Stralberg , A. Hamann , B. H. Mcrae , T. Wang , Global Change Biol. 2017, 23, 4508. 10.1111/gcb.13679 28267245

[76] X. Deng , C. Zhao , H. Yan , Adv. Meteorol. 2013, 2013, 1375.

[77] G. Jin , P. Wang , T. Zhao , Y. Bai , C. Zhao , D. Chen , Phys. Chem. Earth 2015, 89–90, 33.

[78] P. Vihervaara , A.‐P. Auvinen , L. Mononen , M. Törmä , P. Ahlroth , S. Anttila , K. Böttcher , M. Forsius , J. Heino , J. Heliölä , M. Koskelainen , M. Kuussaari , K. Meissner , O. Ojala , S. Tuominen , M. Viitasalo , R. Virkkala , Global Ecol. Conserv. 2017, 10, 43.

